# Revision of the *Orbamia* Herbulot, 1966 group of genera with description of two new genera, ten new species, and two new subspecies (Lepidoptera, Geometridae, Ennominae, Cassymini)

**DOI:** 10.3897/zookeys.929.50391

**Published:** 2020-04-22

**Authors:** Tesfu Fekensa Tujuba, Axel Hausmann, Andrea Sciarretta

**Affiliations:** 1 Ethiopian Biodiversity Institute, Comoros street, Addis Ababa, Ethiopia; 2 SNSB-Zoologische Staatssammlung München, Münchhausenstr, 21, Munich, Germany; 3 Department of Agriculture, Environment and Food Sciences, University of Molise, Via Francesco De Sanctis Campobasso, Italy

**Keywords:** Africa, DNA Barcoding, geometrid moths, integrative taxonomy, Lepidoptera, *
Morabia
*, *
Rabomia
*, species description

## Abstract

The genus *Orbamia* Herbulot, 1966 is revised. Two new genera are described: *Rabomia* Hausmann & Tujuba, **gen. nov.** (type species: Ectropis ? subaurata Warren, 1899), and *Morabia* Hausmann & Tujuba, **gen. nov.** (type species: *Morabia politzari* Hausmann & Tujuba, **sp. nov.**). Ten new species and two new subspecies are described: *Rabomia obscurior* Hausmann & Tujuba, **sp. nov.**, from western Africa, *Morabia politzari* Hausmann & Tujuba, **sp. nov.**, from Kenya, *Morabia brunnea* Hausmann & Tujuba, **sp. nov.**, from Zambia, *Orbamia marginata* Hausmann & Tujuba, **sp. nov.**, from Tanzania, *Orbamia clarissima* Hausmann & Tujuba, **sp. nov.**, from Kenya, *Orbamia clarior* Hausmann & Tujuba, **sp. nov.**, from Kenya, *Orbamia obliqua* Hausmann & Tujuba, **sp. nov.**, from Zambia, *Orbamia obliqua parva* Hausmann & Tujuba, **subsp. nov.**, from South Africa, *Orbamia abiyi* Hausmann & Tujuba, **sp. nov.**, from Zambia, Tanzania, Ethiopia, *Orbamia emanai* Hausmann & Tujuba, **sp. nov.**, from Ethiopia, *Orbamia emanai lenzi* Hausmann & Tujuba, **subsp. nov.**, from Zambia and Malawi, and *Orbamia balensis* Hausmann & Tujuba, **sp. nov.** from Ethiopia. The taxon *Lepiodes ocellata* Warren, 1897 is raised from synonymy of *O. octomaculata* (Wallengren, 1872) to species rank (Zambia, Tanzania, Rwanda). The taxonomical analysis is based on both morphological and genetic cytochrome oxidase I (COI) data. Adults and male and female genitalia of all species are illustrated.

## Introduction

After 260 years of intensive work, taxonomists worldwide have together achieved the formal descriptions of approximately 160,000 lepidopteran species (Van Nieukerken et al. 2011), of which some 24,000 are geometrid moths (cf. Scoble 1999; Scoble and Hausmann 2007; Van Nieukerken et al. 2011). Many more are likely to be described and some recent revisions of many genera revealed high percentages of undescribed species (e.g., Brehm 2015, 2018; Hausmann 2003; Hausmann et al. 2016). This probably covers less than half the number of actual extant geometrid species on earth, an estimate which can be inferred from apparently undescribed species in natural history museums and in molecular databases like the Barcode of Life Data Systems (BOLD: Ratnasingham and Hebert 2007). We conclude that conventional taxonomy with an actual description rate of 80–100 species per year works too slowly for addressing the biodiversity of our Earth (taxonomic impediment: de Carvalho et al. 2007; Wheeler 2008). In these times where we are facing a serious extinction rate we cannot afford to wait for another 250–600 years for the taxonomic assessment of our biodiversity. In the recent literature, several pleas for an accelerated taxonomy have been made (Riedel et al. 2013a; Forum Herbulot 2014) and some taxonomists already published exemplary revisions with shortened descriptions (Riedel et al. 2013b; Meierotto et al. 2019).

In this taxonomic revision we follow such a model of accelerated taxonomy (cf. Riedel et al. 2013a, 2013b; Forum Herbulot 2014; Meierotto et al. 2019), which should lead in future to an automated, easy and rapid transfer of genetic data, images and metadata directly from BOLD into manuscripts and which will allow for continuous updates. Similarly, all nomenclatorial information (valid names, synonyms, original descriptions with authorship and year, type localities, type specimens and their deposition) may in future be transferred in an automated way either from BOLD, from the Global Lepidoptera checklist (Banki et al. 2019) and/or from the Geometridae Mundi database once it will be completed by the Forum Herbulot initiative (cf. Löbel and Hausmann 2019). Similar to the approach of Meierotto et al. (2019) we believe that DNA barcodes in most cases are an excellent tool for species diagnosis but that all descriptions should be linked with a brief description of characters in words, supplemented with photographs. In future, such revisions may also be organised in a flexible way, i.e., with the possibility to subsequently publish updated versions (keeping the previous versions visible) with more data, newly added species, and revised taxonomic concepts. In the framework of the ongoing research project “GBOL III - Dark Taxa” (SNSB – Bavarian State Collection of Zoology, Munich) similar workflows are planned to be tested and established.

The genus *Orbamia* Herbulot, 1966 is restricted to the Ethiopian region, where it was so far represented by five species (Scoble 1999; Hausmann 2006): *Orbamia octomaculata* (Wallengren 1872), *Orbamia pauperata* Herbulot, 1966, *Orbamia renimacula* (Prout, 1926), *Orbamia subaurata* (Warren, 1899) and *Orbamia becki* Hausmann, 2006. Although the external appearance of *Orbamia* is somewhat reminiscent of that of the tribe Boarmiini, Hausmann (2006) placed *Orbamia* in the tribe Cassymini, due to the long process extending from the base of the dorsal margin of the male genital valvae, similar to the equivalent present in *Zamarada*. Molecular evidence for this tribal assignment was given by Murillo-Ramos et al. (2019) and Brehm et al. (2019). Species of *Orbamia* are recognised by the following characters: wings with conspicuous, contrasting discal spots on all wings, antennae bipectinate in males and filiform in females, dorsal process in male genitalia strongly curved.

## Materials and methods

In the present paper, the material housed in the Zoologische Staatssammlung München (**ZSM**), Munich, Germany, collected from 22 different African and Arabian countries, has been studied. Two relevant type specimens available in the ZSM and two from the Natural History Museum in London (**NHMUK**) were examined. Altogether, 298 specimens of the genera *Orbamia*, *Rabomia* gen. nov., and *Morabia* gen. nov. have been examined.

Comparative morphological methods and COI sequence divergences were used to delimit the taxa and to estimate their taxonomic status. We studied morphological characters of adults (including genitalia and wing venation). The abdomens and genitalia were prepared using the method of Hardwick (1950). The analysis is furthermore based on 58 genitalia slides and 72 DNA barcodes.

For DNA analyses, one or two legs were removed from dried specimens and stored in an individual tube, in absolute ethanol. DNA extraction, amplification and sequencing of the “barcode” region of the mitochondrial cytochrome c oxidase I (COI) gene region (658 base pairs) were carried out in the Canadian Centre for DNA Barcoding, Ontario, Canada, using standard high through-put protocols (Ivanova et al. 2006). Sequence divergences within and between species were calculated using the Kimura 2-parameter model (Kimura 1980), using the analytical tools provided by BOLD Systems v4 platform (Ratnasingham and Hebert 2007; http://www.boldsystems.org/). Intra-specific and inter-specific genetic distances are reported as maximum and minimum distances, respectively. The Barcode Index Number (BIN) of each species is reported which was obtained from the BOLD Systems v4 database. BINs represent a species-level taxonomic registry of the animal kingdom based on the analysis of nucleotide variation patterns in the barcode region of the cytochrome c oxidase I (COI) gene (Ratnasingham and Hebert 2013). This genetic information facilitates the species delimitation and constitutes the basis of future phylogenetic works at the genus level and below (Brehm 2015, 2018).

Label data and photographs of types and other barcoded specimens are accessible on BOLD, dataset DS-ORBAMIA (https://dx.doi.org/10.5883/DS-ORBAMIA). All new names are registered in ZooBank. Geo-references were taken from specimen labels.

## Systematic accounts

### Orbamia

Taxon classificationAnimaliaLepidopteraGeometridae

Herbulot, 1966

4630FAE0-1BE5-5CDF-9726-A60D0171E931

Herbulot (1966): 221. Type species: Orbamia octomaculata Wallengren, 1872

#### Differential features

(COI sequences, photographs of adults and their genitalia see https://dx.doi.org/10.5883/DS-ORBAMIA). Adult: Male antennae bipectinate. Upperside of wings with conspicuous, contrasting discal spots on all wings. Underside with yellowish scales, with darker pattern towards termen. Male genitalia: Differing from those of the other two genera by longer uncus, naked dorsal process of valva, long and narrow cornutus. Female genitalia: Apophyses anteriores usually half-length of apophyses posteriores, lamellae ante- and post-vaginalis sclerotised (often oval), ductus bursae membranous, signum a small sclerite with transverse ridge.

#### Genetic data and phylogeny.

Multigene analyses of Geometridae revealed evidence for assignation of the genus *Orbamia* to Cassymini and for sister group relationship with the African genus *Pycnostega* (Murillo-Ramos et al. 2019; Brehm et al. 2019). The maximum likelihood analysis of COI barcode data underpins the monophyly of the genus *Orbamia* as conceived and circumscribed here (cf. Table 1, Fig. 85).

**Table 1.** Barcode gap analysis of COI data of the genera *Orbamia*, *Rabomia*, and *Morabia* (https://dx.doi.org/10.5883/DS-ORBAMIA), showing intraspecific variation (“Mean Intra-Sp” and “Max. Intra-Sp.”) and distances from Nearest Neighbour Species (“NN”).

Species	Mean Intra-Sp	Max. Intra-Sp	Nearest species	Distance NN
*Orbamia abiyi*	0.51	0.93	*Orbamia clarior*	2.1
*Orbamia balensis*	0.61	0.61	*Orbamia ocellata*	7.65
*Orbamia becki*	0.97	2.68	*Orbamia marginata*	1.89
*Orbamia clarior*	0	0	*Orbamia abiyi*	2.1
*Orbamia ocellata*	0	0	*Orbamia clarior*	2.81
*Orbamia emanai*	1.43	2.17	*Orbamia clarior*	2.47
*Orbamia marginata*	0.53	0.62	*Orbamia becki*	1.89
*Orbamia obliqua*	1.35	1.87	*Orbamia abiyi*	3.63
*Orbamia octomaculata*	0.69	1.27	*Orbamia marginata*	2.34
*Orbamia renimacula*	0.42	1.08	*Orbamia becki*	2.18
*Rabomia obscurata*	N/A	0	*Rabomia subaurata*	5.19
*Rabomia subaurata*	0.41	0.62	*Rabomia obscurata*	5.19
*Morabia brunnea*	N/A	0	*Morabia politzari*	2.66
*Morabia politzari*	0.25	0.26	*Morabia brunnea*	2.66

### Orbamia octomaculata

Taxon classificationAnimaliaLepidopteraGeometridae

(Wallengren, 1872)

24691D09-8FFB-58DF-ADA5-6048C7F77075

BIN: BOLD: AAQ4039

**Figures 1–18.**
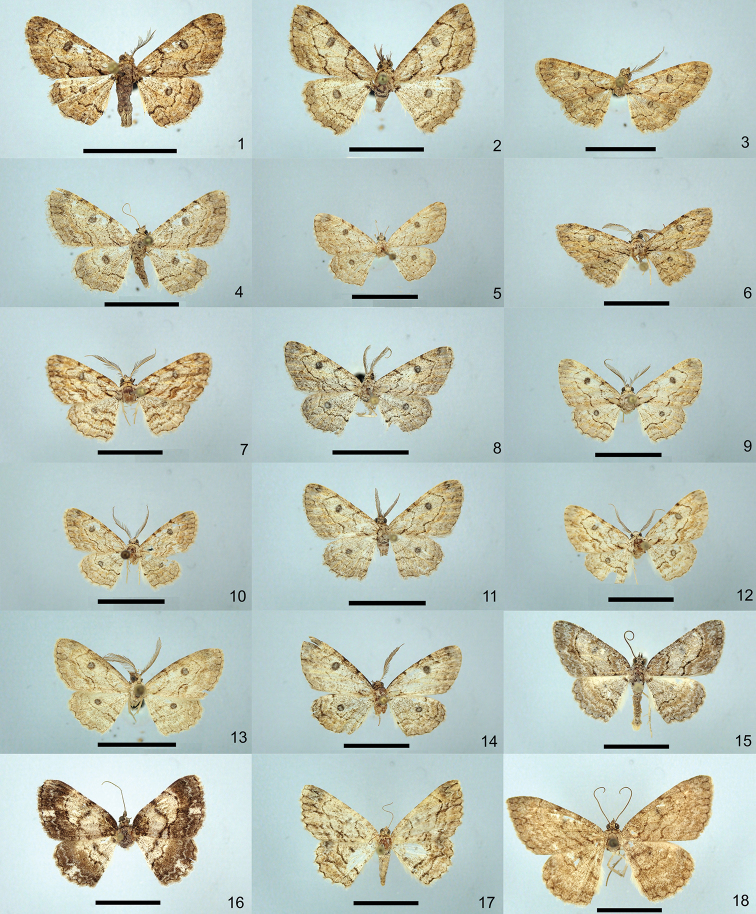
Specimens of the genera *Orbamia*, *Rabomia*, and *Morabia*, dorsal view. **1***Orbamia octomaculata***2***O. marginata* (paratype) **3***O. becki* (holotype) **4***O. renimacula***5***O. clarissima* (holotype) **6***O. clarior* (holotype) **7***O. obliqua* (holotype) **8***O. obliqua parva* (holotype) **9***O. ocellata***10***O. abiyi* (holotype) **11***O. emanai* (holotype) **12***O. emanai lenzi* (holotype) **13***O. pauperata* (holotype) **14***O. balensis* (holotype) **15***Rabomia subaurata***16***R. obscurior* (holotype) **17***Morabia politzari* (holotype) **18***M. brunnea* (holotype). Scale bars: 1 cm.

**Figures 19–27.**
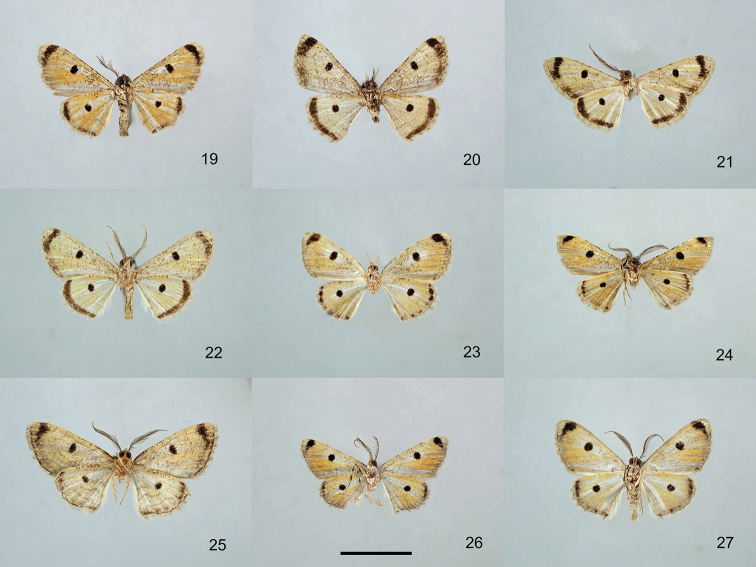
Specimens of the genus *Orbamia*, ventral view. **19***Orbamia octomaculata***20***O. marginata***21***O. becki***22***O. renimacula***23***O. clarissima***24***O. clarior***25***O. obliqua***26***O. obliqua parva***27***O. ocellata*. Scale bars: 1 cm.

Figures 1 , 19 , 37 , 55 , 73

Panagra octomaculata : Wallengren (1872): 60 (Holotype ♂ in NHRS, Stockholm; locus typicus: [South Africa]: “Caffraria orientalis interior“)

#### Examined material (ZSM).

25♂♀ from Tanzania, Zambia, Botswana, Mozambique, Malawi, Namibia, South Africa (ZSM G 20929/♀; ZSM G 20921/♂; ZSM G 20930/♂; ZSM G 20931/♂; ZSM G 20943/♂; ZSM G 13646/♂; ZSM G 13645/♀; ZSM G 20947/♀).

#### Differential features

(COI sequences, photographs of adults and their genitalia see https://dx.doi.org/10.5883/DS-ORBAMIA): Adult: Forewing length: 11.5–13.5 mm. Upperside of wings: Ground colour pale brown with dark suffusion. Underside: Ground colour yellow or orange with much dark suffusion, terminal fascia on hind wing usually complete, on forewing restricted to apex. Male genitalia: Uncus long, triangular, valva strongly (rectangularly) bent, dorsal process with small spinule at tip, cornutus short and stout (1.4–1.7 mm). Female genitalia: Lamella antevaginalis U-shaped, long (0.75 mm), signum sclerotised, transversely flat, transverse ridge curved (0.4–0.5 mm).

### Orbamia marginata

Taxon classificationAnimaliaLepidopteraGeometridae

Hausmann & Tujuba sp. nov.

5A204640-055E-5450-B867-A6B29006E797

http://zoobank.org/6B79EAB5-4CE5-4B46-9176-5A47FF51E0AB

BIN: BOLD: AAZ5266

Figures 2 , 20 , 38 , 56

#### Examined material.

***Holotype***: ♂, Tanzania, Bukwa region, 14 km W Namanyere, 1290 m, 07°27.28'S, 030°54.49'E, 14.xi.2005, leg. Ph. Darge, coll. ZSM (ZSM G 20909).

***Paratypes***: 2♂, Tanzania, Rukwa region, 14 km W Namanyere, 1290 m, 07°27.28'S, 030°54.49'E, 14.xi.2005, leg. Ph. Darge, coll. ZSM (ZSM G 20941); 1♂, Tanzania, Rukwa region, Luafi Game Reserve (W. Namanyere), 1260 m, 07°26.98'S, 030°54.24'E, 31.i.2008, leg. Ph. Darge; 1♂, Tanzanie, Iringa region, Iyayi savanna, 1400 m, 08°51.47'S, 034°31.29'E, 14.iv.2007, Ph. Darge; 1♂, Tanzania, Morogoro region, Udzungwa N.P. camp site, 3315 m, 07°50.95'S, 036°50.95'E, 26.xi.2005, leg. Ph. Darge (all ZSM).

#### Etymology.

The name refers to the uninterrupted black line at the hindwing margin (Lat. margo/marginis = edge, border).

#### Differential features

(COI sequences, photographs of adults and their genitalia see https://dx.doi.org/10.5883/DS-ORBAMIA): Adult: Forewing length: 11.5–12.5 mm. Upper side of wings: Ground colour dirty grey with brown suffusion. Underside: Ground colour whitish beige with some dark suffusion, terminal fascia conspicuous, uninterrupted on all wings. Male genitalia: Uncus long, triangular, valva slightly bent, dorsal process with small spinule at tip, cornutus short and stout (1.6 mm). Female genitalia unknown.

### Orbamia becki

Taxon classificationAnimaliaLepidopteraGeometridae

Hausmann, 2006

B15B0A09-BEDF-59B0-A95B-3BDE5DCB0130

BIN: BOLD: AAD8768

Figures 3 , 21 , 39 , 57 , 74

Orbamia becki : Hausmann (2006): 42 (Holotype ♂ in ZSM: G 13627; locus typicus: Yemen: Al Hudaydah, Jebel Burra).

#### Examined material (ZSM).

57♂♀ from Yemen, Ethiopia and Djibouti (ZSM G 13478/♂; ZSM G 13628/♀; ZSM G 13649/♀; ZSM G 13636/♂; ZSM G 13637/♀/ ZSM G 13650/♀; ZSM G 20927/♀).

#### Differential features

(COI sequences, photographs of adults and their genitalia see https://dx.doi.org/10.5883/DS-ORBAMIA): Adult: Forewing length: 9.5–12 mm. Upperside of wings: Ground colour pale brown. Underside: Ground colour whitish with yellowish tinge, on forewing with some dark suffusion, terminal fascia on all wings complete. Male genitalia very similar to those of *O. octomaculata*: Uncus stout, triangular, valva strongly (rectangularly) bent, dorsal process with small spinule at tip, cornutus short and stout (1.5 mm). Female genitalia very similar to those of *O. octomaculata*: Lamella antevaginalis U-shaped (0.6–0.7 mm), signum sclerotised, transversely oval, transverse ridge curved (0.3–0.5 mm).

#### Remarks.

Allopatric vicariant of *O. octomaculata*. *Orbamia becki* is the only species of this genus that has also been recorded outside Africa in Yemen, southern Arabia.

### Orbamia renimacula

Taxon classificationAnimaliaLepidopteraGeometridae

(Prout, 1926)

FF99252D-A5E0-5284-8205-22E01E0AF9D5

BIN: BOLD: AAE1536

Figures 4 , 22 , 40 , 58 , 75

Boarmia renimacula : Prout (1926): 184 (holotype ♀ in NHMUK; locus typicus: Senegal: Sédhiou).

#### Examined material (ZSM).

45♂♀ from Cameroon, Burkina Faso (Upper Volta), Guinea, Senegal, Togo, Mali, Gambia, Nigeria and Ivory Coast (ZSM G 13626/♂; ZSM G 61321/♀; ZSM G 13620/♂).

#### Differential features

(COI sequences, photographs of adults and their genitalia see https://dx.doi.org/10.5883/DS-ORBAMIA): Adult: Forewing length: 8.5–12 mm, one of the smallest *Orbamia* species. Upperside of wings: Ground colour pale grey with brown suffusion. Underside: Ground colour white with very slight yellowish tinge, and slight dark suffusion, terminal fascia on all wings complete, conspicuous, at forewing apex dilated. Male genitalia: Uncus narrow digitiform, saccus round, valva almost straight, dorsal process with fine, curved hook at tip, cornutus of medium length (2.0 mm). Female genitalia: Lamella post-vaginalis weakly sclerotised, lamella ante-vaginalis U-shaped (0.6 mm), signum sclerotised, rhomboid, transverse ridge straight (0.25 mm).

### Orbamia clarissima

Taxon classificationAnimaliaLepidopteraGeometridae

Hausmann & Tujuba sp. nov.

72976112-881A-5A25-AF7C-1ADE9F8D1391

http://zoobank.org/38E93700-3258-4497-B742-39B80BB75B0D

BIN: not yet assigned, DNA barcodes BC ZSM Lep 106553, 106554, 106555.

Figures 5 , 23 , 41 , 59 , 76

#### Examined material.

***Holotype***: ♀, Kibwezi, B.E.A. [Kenya], 12 March 1917 (W. Feather), coll. ZSM (ZSM G 13618).

***Paratypes***: 1♂, Kibwezi, B.E.A. [Kenya], April 1922 (W. Feather); 1♂, Kenya, Mutha, 5.IV.69, Watulege; 1♀, Kenya, Musthomo, 13.III.69, Watulege (ZSM G 13617); 1♂, Somalia m., Caonole Fluß, 21.1.1988, leg. Dr. Politzar (all ZSM). ZSM G 13647/♂.

#### Etymology.

The name refers to the very pale ground colour (Lat. clarissimus, -a, -um = palest, clearest).

#### Differential features

(COI sequences, photographs of adults and their genitalia see https://dx.doi.org/10.5883/DS-ORBAMIA): Adult: Forewing length: 7.5–11 mm, one of the smallest *Orbamia* species. Upperside of wings: Ground colour whitish with slight grey brown suffusion. Underside: Ground colour white with slight orange tinge, mainly towards termen between veins, apical spots on all wings conspicuous, sharp. Male genitalia: Uncus long, digitiform, stout, valva straight, dorsal process with small spinule at tip, cornutus very short (1.0 mm) and tiny. Female genitalia: Apophyses stout, apophyses anteriores comparatively long (2/3 length of apophyses posteriores), lamella ante-vaginalis heart-shaped (length and width 0.45 mm), signum small, sclerotised, transverse ridge straight (0.15 mm).

### Orbamia clarior

Taxon classificationAnimaliaLepidopteraGeometridae

Hausmann & Tujuba sp. nov.

2A5C9C11-7810-5B9F-9728-B87E3F4078F2

http://zoobank.org/79B39635-880A-411F-B9C8-563320CA5766

BIN: BOLD: ABW5825

Figures 6 , 24 , 42 , 60

#### Examined material.

Holotype: ♂, Kenya, South Ukanbasi, 6.V.1996, leg. Politzar (ZSM G 20944).

#### Etymology.

The name refers to the comparatively pale ground colour.

#### Differential features

(COI sequences, photographs of adults and their genitalia see https://dx.doi.org/10.5883/DS-ORBAMIA): Forewing length: 10 mm. Upperside of wings: Ground colour comparatively dark brownish, transverse lines of forewing oblique. Underside: Ground colour whitish beige, orange between veins, apical spot on forewing conspicuous, sharp, on hindwing narrow, elongate, remnants of dark colouration in the anal angle. Male genitalia: Uncus of medium length, digitiform, stout, dilated towards base, valva straight, dorsal process with conspicuous spinule at tip, cornutus very long (2.7 mm). Female genitalia unknown.

### Orbamia obliqua

Taxon classificationAnimaliaLepidopteraGeometridae

Hausmann & Tujuba sp. nov.

8D0D1090-515C-50F8-9F7F-378F15965821

http://zoobank.org/BED6B29E-D967-419C-A998-6773D7BF078D

BIN: BOLD: AAM4892

Figures 7 , 25 , 43 , 61

#### Examined material.

***Holotype***: ♂, North-western Zambia, Hillwood farm, 11°16.01'S, 24°18.99'E, 17.ix.2009, 1420 m, UV, J. Lenz legit, coll. ZSM (G 20905).

***Paratype***: 1♂, Tanzania, Morogoro province, Nguru mounts, IV.2004 (ex coll. Philippe Darge, coll. ZSM, G 20906).

#### Etymology.

The name refers to the oblique position of the transverse lines of the forewing (Lat. obliquus, -a, -um = oblique).

#### Differential features

(COI sequences, photographs of adults and their genitalia see https://dx.doi.org/10.5883/DS-ORBAMIA): Adult: Forewing length: 11–12 mm. Upperside of wings: Ground colour pale grey with brown pattern, transverse lines oblique. Underside: Ground colour beige with slight yellowish tinge, and with strong dark suffusion, terminal area with pattern reduced to a dark apical spot and a dark shadow on the hindwing apex. Male genitalia: Uncus short, stout, hook-shaped, saccus projection shallow, valva straight, narrow at tip, dorsal process with a stout hook at tip, cornutus of medium length (1.9 mm). Female genitalia unknown.

### Orbamia obliqua parva

Taxon classificationAnimaliaLepidopteraGeometridae

Hausmann & Tujuba subsp. nov.

1FB2E729-91EF-502C-AD9D-35A48A5D6497

http://zoobank.org/3A0A5166-35FB-4D67-9BC2-B4C5C1024779

BIN: BOLD: AAM4892

Figures 8 , 26 , 44 , 62

#### Examined material.

***Holotype***: ♂, South Africa, Limpopo, Melkrivier Lapalala, Wilderness Kolobe camp, 1220 m -23.9094/28.2736, 13.xi.2017, leg. A. Hausmann, coll. Ditsong Museum, Pretoria, gen. prp. ZSM G 20933.

***Paratypes***: 6♂, South Africa, Limpopo, Melkrivier Lapalala, Wilderness, Kolobe camp, 1220 m -23.9094/28.2736, 13.xi.2017, leg. A. Hausmann; 2♂, RSA, Northwest prov. 7.5 km North Zeerust, 1180 m (lux), 25°27'S, 26°05'E, 17.II.2006, leg. Hacker (ZSM G 20949); 1♂, South Africa, Gauteng, Mogale’s Gate Biodiversity Centre, near Bush Camp, -25.938, 27.639, 1420 m, 14.ii.2012, leg. P. Hebert, J. deWaard, coll. University of Guelph (Canada), Centre for Biodiversity Genomics.

#### Etymology.

The name refers to the small size of this subspecies (Lat. parvus, -a, -um = small), being much smaller than the sympatric *O. octomaculata*.

#### Differential features

(COI sequences, photographs of adults and their genitalia see https://dx.doi.org/10.5883/DS-ORBAMIA): Adult: Forewing length: 9–11 mm. Upperside of wings: Ground colour pale grey, darker in the terminal area, pattern dark grey. Underside: Ground colour whitish beige, orange between veins, on forewing apex a sharp black spot, dark colouration on the hindwing terminal area restricted to a small stripe or shadow in the apex. Male genitalia: Uncus comparatively short, stout, hook-shaped, saccus projection shallow, valva straight, narrow at tip, dorsal process with a stout hook at tip, cornutus of medium length (1.6–1.9 mm). Female genitalia unknown.

### Orbamia ocellata

Taxon classificationAnimaliaLepidopteraGeometridae

(Warren, 1897) stat. nov.

B5CF2323-98C7-5922-9C06-3FBAD9B25B28

BIN: BOLD: AAP8312

Figures 9 , 27 , 45 , 63 , 77

Lepiodes ocellata : Warren (1897): 94 (Syntypes 5♂1♀ in NHMUK; locus typicus: South Africa: Bathurst; [north-eastern Zambia]: Mpeta, Loangwa River [Luangwa], off the Zambesi [close to the border with northern Malawi]).

#### Note.

Synonym of *O. octomaculata* according to Scoble (1999) but the type series of *ocellata* belongs to two different species. Herewith we designate the red-ring-labelled male specimen from Zambia, Mpeta, as lectotype to fix the identity of the name and to stabilise nomenclature. The taxon is herewith upgraded from synonymy to species rank (stat. nov.), based on the below mentioned differences in DNA barcodes and genitalia. Pattern of upper- and underside of wings of the lectotype exactly matches that of the examined material from Tanzania, partly collected in closely adjacent localities to the type locality (e.g., Ruvuma and Iringa provinces).

#### Examined material.

1♂, Tanzania, Pwani region, Manadera, 166m, 06°14.30'S, 038°23.19'E, 07.XII.2008, leg. Ph. Darge (ZSM G 20908); 1♀, Tanzanie, Tanga region, Savane prės, Usambara west. 475 m, 11.v.2005 leg. Ph. Darge; 2♀, Tanzanie, Tanga region, West Usambara mts, Magamba Forest, 1818 m, 04°42.76'S, 038°17.28'E, 01.XII.2008, leg. Ph. Darge; 1♀, Tanzanie, Morongoi region, Mikesse Hills, 375 m, 06°40.50'S, 037°58.12'E, 17.XI.2004, leg. Ph. Darge; 2♀, Tanzanie, Morogoro 1 km E Mikumi, 550 m, 5.III.2003, leg. M. Fibiger, H. Hacker, K.Larsen, H.P. Schreier; 1♀, Tanzania, Morogoro region, Uluguru mts, Bunduki Forest, 1275 m, 07°01.07'S, 037°37.94'E, 23.XI.2007, leg. Ph. Darge; 1♀, Tanzania, Morogoro region, face West des, Nguru mts, Makuyu, savane arborėe, 620 m, 25.IV.2005, leg. Ph. Darge (ZSM G 20942); 2♀, Tanzania, Morogoro region, West Nguru mts, Makuyu, alt. 620 m, 06°16.08'S, 037°20.54'E, 19.XI.2007, leg. Ph. Darge; 1♂, Tanzania, Rukwa prov, Kisengere/ Kasambo, 1193 m, 07°27.54'S, 030°52.81'E, 17.v.2004, leg. Ph. Darge; 1♂, Tanzania, Rukwa prov., Mbizi mts, entre Kisungu et Muze, 1415 m, 07°43.82'S, 031°32.4'E, 14.v.2004, leg. Ph. Darge; 1♀, Tanzanie, Iringa, Ulembwa, 2070 m, 09°18.70'S, 034°38.07'E, 22.XII.2008, leg. Ph. Darge (ZSM G 20911); 2♀, Tanzanie, Pwani region, Savane de Mandera, 170 m, 06°14.30'S, 038°23.19'E, 19.III.2006, leg. Ph. Darge; 1♂2♀, id., 15.I.2005; 1♀, Tanzanie, Ruvuma region, Kitai Savnna, 1020 m, 10°42.40'S, 035°12.33'E, 24.III.2006, leg. Ph. Darge (ZSM G 20946); 1♂, Tanzanie, Mbeya region, Igurusi savanna, 1150 m, 08°46.68'S, 033°46.17'E, 06.IV.2006, leg. Ph. Darge; 3♀, Tanzania, Ubenazomizi region, savannas and deciduous forest, 450 m, 06°40.57'S, 037°58.99'E, 13.XII.2002, leg. Ph. Darge and Th. Ebode; 1♀, Tanzanie, Ruvuma region, Kitai Savanna, 1020 m, 10°42.40'S, 035°12.33'E, 24.III.2006, leg. Ph. Darge.

#### Differential features

(COI sequences, photographs of adults and their genitalia see https://dx.doi.org/10.5883/DS-ORBAMIA): Adult: Forewing length: 8.5–12 mm. Upperside of wings: Ground colour pale grey with slight brown suffusion, pattern brown with slight orange tinge. Underside: Ground colour whitish, orange between veins, apical spots on forewing conspicuous, sharply bordered, on hindwing terminal fascia usually diffuse, rarely restricted to apex. Male genitalia: Uncus narrow, digitiform, valva straight, broad, dorsal process with conspicuous, stout hook at tip, cornutus narrow and very long (2.8–3.0 mm). Female genitalia: Apophyses stout, apophyses anteriores comparatively long (2/3 length of apophyses posteriores), lamella ante- and post-vaginalis fused, oval, comparatively broad (length 0.7 mm, width 0.5 mm), signum weakly sclerotised, small, transverse ridge straight (0.2 mm).

### Orbamia abiyi

Taxon classificationAnimaliaLepidopteraGeometridae

Hausmann & Tujuba sp. nov.

9787AB5E-CEF9-53E3-877D-25024A1B1390

http://zoobank.org/8AC03FE5-C57D-4BA1-9C63-44281F0AB521

BIN: BOLD: AAK5536

**Figures 28–36.**
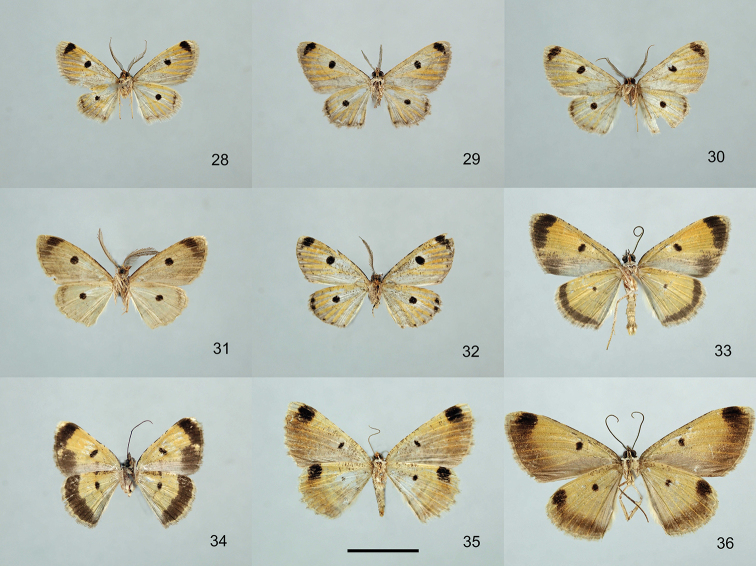
Specimens of the genera *Orbamia*, *Rabomia*, and *Morabia*, ventral view. **28***Orbamia abiyi***29***O. emanai***30***O. emanai lenzi***31***O. pauperata***32***O. balensis***33***Rabomia subaurata***34***R. obscurior***35***Morabia politzari***36***M. brunnea*. Scale bars: 1 cm.

Figures 10 , 28 , 46 , 64 , 78

#### Examined material.

***Holotype***: ♂, North Zambia, Mutinondo, 1390 m, wet Miombo, 29.XII. 2010, 12°23'30"S, 31°19'23"E, light trap, J. Lenz legit (ZSM G 20912).

***Paratypes***: 1♂1♀, North Zambia, Mutinondo, 1390 m, wet Miombo, 29.XII.2010, 12°23'30"S, 31°19'23"E, light trap, J. Lenz legit (ZSM G 20913/♀); 1♂, S. Ethiopia SN, Arba Minch, below Hotel Bekele Molla, thornbush, 1310 m, 10°26'N, 39°53'E, 29.IV.2008, leg. Hacker & Schreier (ZSM G 20556); 1♂, Tanzania, Morogoro region, Mikesse Hills, 420 m, 06°40.15'S, 037°57.57'E, 21.XII.2005, Ph. Darge; 1♀, Tanzania, Rukwa prov., Mbizi mts, entre Kisungu et Muze, 1415 m, 07°43.82'S, 031°32.48'E, 14.v.2004, leg. Ph. Darge; 1♂, Namibia, Kavango distr., 17°52'N, 19°39'E, 16 km W Rundu, (Okavango) Kavango river area, 28.II.2006, leg. H. Hacker & H.P. Schreier (ZSM G 20932); 2♂, Rwanda, S.E. Rusumo, 1300 m, 29.XII.1975, leg. B. Turlin (ZSM G 13625; BC ZSM Lep) ; 1♂, id., 25.3.1975 (ZSM).

#### Etymology.

The name honours his Excellency Dr Abiy Ahmed Ethiopia’s Prime Minister, the 2019 Nobel Peace Prize Laureate, for his tremendous contributions to Ethiopia and the Horn of African geopolitics.

#### Differential features

(COI sequences, photographs of adults and their genitalia see https://dx.doi.org/10.5883/DS-ORBAMIA): Adult: Forewing length: 10–12 mm. Upperside of wings: Ground colour pale grey with slight brown suffusion, pattern brown with very slight orange tinge. Underside: Ground colour whitish, orange between veins, apical spots on forewing conspicuous, sharply bordered, on hindwing terminal fascia usually diffuse, in Tanzania hindwing terminal fascia uninterrupted. Male genitalia: Uncus narrow, digitiform, valva straight, broad, dorsal process with conspicuous, stout hook at tip, cornutus narrow and long (2.2–2.3 mm). Female genitalia: Lamella ante- and post-vaginalis fused, oval (length 0.75–0.85 mm), signum weakly sclerotised, small, transverse ridge straight (0.17–0.2 mm).

#### Remarks.

Populations from western Ethiopia with darker upperside of wings, on hindwing underside dark pattern reduced to apex. See also remarks to the following species.

### Orbamia emanai

Taxon classificationAnimaliaLepidopteraGeometridae

Hausmann & Tujuba sp. nov.

C7825797-EEDF-54B1-BFE1-D040A374ADF1

http://zoobank.org/5A4DFBCF-0D52-4378-AF90-84611329CBAE

BIN: BOLD: ABW6858

Figures 11 , 29 , 47 , 65 , 79

#### Examined material.

***Holotype***: ♂, NW Ethiopia, 30 km SE Bahir Dar, Tis Isat, Blue Nile falls, 1640 m, 11°29'08"N, 37°35'28"E, 25.VI.2008, leg. Hacker & Schreier (ZSM G 20917).

***Paratypes***: 30♂8♀, NW Ethiopia, 30 km SE Bahir Dar, Tis Isat, Blue Nile falls, 1640 m, 11°29'08"N, 37°35'28"E, 25.VI.2008, leg. Hacker & Schreier; 1♂, S. Ethiopia S.N., 12 km W Jinka, border of Mago N.P., 930 M (lux), 05°18'47"N, 36°44'07"E, 6.V.2008, leg. Hacker & Schreier (ZSM G 20910/♀; ZSM G 20916/♀).

#### Additional material

(exactly barcode-sharing): 8♂2♀, Botswana, Central distr. 15 km NW Francistown, river Tati, 1030 m, 21°02'S, 27°32'E, 19.II.2006, Hacker & Schreier (ZSM G 20919/♀; ZSM G 20920/♂); 1♀, Botswana, Kgatleng distr., 30 km NNE Gaborone, 980 m (lux), 19.II.2006, leg Hacker & Schreier; 2♂, Botswana, Central district 10 km SSE Nata, Sua pan, 930 m (lux) 20°09'S, 26°26'E, 20.II.2006, leg Hacker & Schreier.

#### Etymology.

Named after Emana Getu, a senior professor of Entomology, at Addis Ababa University for his immense contributions to the field of entomology.

#### Differential features

(COI sequences, photographs of adults and their genitalia see https://dx.doi.org/10.5883/DS-ORBAMIA): Adult: Forewing length: 9–12 mm. Upperside of wings: Ground colour comparatively dark. Underside: Ground colour pale grey, on hindwing with slight orange tinge, on forewing orange between veins, apical spots on forewing conspicuous, sharply bordered, on hindwing slightly darker at apex only. Male genitalia: Uncus narrow, digitiform, valva straight, broad, dorsal process with conspicuous, stout hook at tip, cornutus narrow and very long (2.5–3.0 mm). Female genitalia: Lamella ante- and post-vaginalis fused, oval (length 0.8–0.85 mm), signum weakly sclerotised, small, transverse ridge straight (0.17–0.2 mm).

#### Remarks.

Morphological differences to the previous species small, but the genetic divergence correlates with darker wing colour, and a few characters in male genitalia (longer cornutus). Distribution areas of both species overlapping.

### Orbamia emanai lenzi

Taxon classificationAnimaliaLepidopteraGeometridae

Hausmann & Tujuba subsp. nov.

A53BC694-6144-5651-9004-B6F9058935DA

http://zoobank.org/0FC250FF-B4AF-4461-8113-2D0CFEF85C5F

BIN: BOLD: ABW6858

Figures 12 , 30 , 48 , 66 , 80

#### Examined material.

***Holotype***: ♂, Zambia, North Zambia, Mutinondo, 1390 m, wet Miombo, 01.I. 2011, 12°23'30"S, 31°19'23"E, light trap, J. Lenz legit, gen. prp. ZSM G 20917 (coll. ZSM G 20918).

***Paratypes***: 1♀, Zambia, North Zambia, Mutinondo, 1390 m, wet Miombo, 01.I.2011, 12°23'30"S, 31°19'23"E, light trap, J. Lenz legit (ZSM G 20922); 1♂, id., 29.XII.2010; 1♂, Zambia, Northwest prov., Chiwona riverine forest, 1330 m, -12.412S, 24.1910E, 12.IX.2015, leg Sàfiàn Szabolcs; 1♂, S. Africa [sic!], S. Malawi, Nsanje distr. 25 km S Blantyre, Mwabvi reserve, 16°39.20'S, 35°03.03'E, 10.XII.2010, 127 m, Ustjuzhanin & Kovtunovich (ZSM G 20907).

#### Etymology.

Named after Jürgen Lenz, Harare - Leipzig, active and experienced researcher and collector of geometrids in Africa, mainly Zambia and Zimbabwe.

#### Differential features

(COI sequences, photographs of adults and their genitalia see https://dx.doi.org/10.5883/DS-ORBAMIA): Adult: Forewing length: 10–11 mm. Upperside of wings: Ground colour much paler than in the nominotypical subspecies, very pale grey, with slight brown suffusion, mainly in the terminal area, pattern grey brown. Underside: Ground colour whitish beige, orange on veins, apical spots on forewing conspicuous, sharply bordered, on hindwing terminal fascia usually diffuse over more or less the whole termen. Male genitalia: Uncus narrow, digitiform, valva straight, broad, dorsal process with conspicuous, stout hook at tip, cornutus narrow and long (2.7 mm). Female genitalia: Lamella ante- and post-vaginalis fused, oval (length 0.75–0.85 mm), signum weakly sclerotised, small, transverse ridge straight (0.17–0.2 mm).

#### Remarks.

BIN-sharing but at 2.2 % distance, much paler than the nominotypical subspecies, but little difference in genitalia.

### Orbamia pauperata

Taxon classificationAnimaliaLepidopteraGeometridae

Herbulot, 1966

11503A83-3363-598F-AB9E-1F5B3EFA2BEA

Figs 13 , 31 , 49 , 67 , 81

Orbamia pauperata : Herbulot (1966): 221 (Holotype § in ZSM: gen. prep. ZSM G 14466; locus typicus: Madagascar: Betioky, southern shore of Tsimanampetsotsa).

#### Note.

So far, without BIN, holotype with short barcode fragment (BC ZSM Lep 81698).

#### Examined material.

2♂3♀ from Madagascar, including holotype (ZSM G 13619/♀).

#### Differential features

(COI sequences, photographs of adults and their genitalia see https://dx.doi.org/10.5883/DS-ORBAMIA): Adult: Forewing length: 10–12 mm. Upperside of wings: Ground colour pale grey. Underside: Ground colour beige, without yellow tinge, apical spot present on forewing. Male genitalia: Uncus digitiform, saccus shortly projecting, round, valva short, broad, tapered at tip, dorsal process stoutly sclerotised, with a conspicuous, very long spine at tip, aedeagus short (1.2 mm), cornutus very short (0.7 mm) and S-shaped at tip. Female genitalia: Lamella ante- and post-vaginalis developed as two separate, narrow, transverse sclerites, posteriorly bilobed, signum rhomboid, transverse ridge straight (0.25 mm).

#### Remarks.

Phylogenetically the most isolated species within this genus, based on large differences in morphology and genetics, the latter, however, just based on a short barcode fragment of the holotype.

### Orbamia balensis

Taxon classificationAnimaliaLepidopteraGeometridae

Hausmann & Tujuba sp. nov.

234537E0-0C9E-541F-B9FC-558231852F2C

http://zoobank.org/:0948AD35-4B39-4FF5-A210-5AAD56A1BE4C

BIN: BOLD: AEA2800

Figures 14 , 32 , 50 , 68

#### Examined material.

***Holotype***: ♂, Ethiopia Oromia, Bale 8 km S. Dolo Mena, 1200 m, IV.2017, leg. R. Beck, coll. ZSM (ZSM G 20914).

***Paratypes***: 2♂2♀, Ethiopia Oromia, Bale 8 km S. Dolo Mena, 1200 m, IV.2017, leg. R. Beck (ZSM G 20948/♀); 1♀, Äthiopien, prov. Oromia, Dolo Mena, 30 km, S., 1080 m (savanna) 06°13.53'N, 39°46.82'E, 14.IV.2019, Robert Beck (ZSM).

#### Etymology.

The name refers to the type locality in the Bale mountains.

#### Differential features

(COI sequences, photographs of adults and their genitalia see https://dx.doi.org/10.5883/DS-ORBAMIA): Adult: Forewing length: 11–13 mm. Upperside of wings: Ground colour comparatively dark, with much dark brown suffusion, pattern not well contrasted. Underside: Ground colour beige, with pale orange tinge on veins, apical spots on forewing conspicuous, sharply bordered, on hindwing slightly darker at apex only, remnants of dotted terminal fascia on all wings. Male genitalia: Uncus stout, very long, saccus broad, valva straight, ´very broad at base, narrow at tip, dorsal process stoutly sclerotised, with conspicuous, stout hook at tip, cornutus narrow and short (1.1 mm). Female genitalia: Lamella ante- and post-vaginalis fused, heart-shaped, short (length 0.6 mm), signum small, transverse ridge straight (0.2 mm).

**Figures 37–45.**
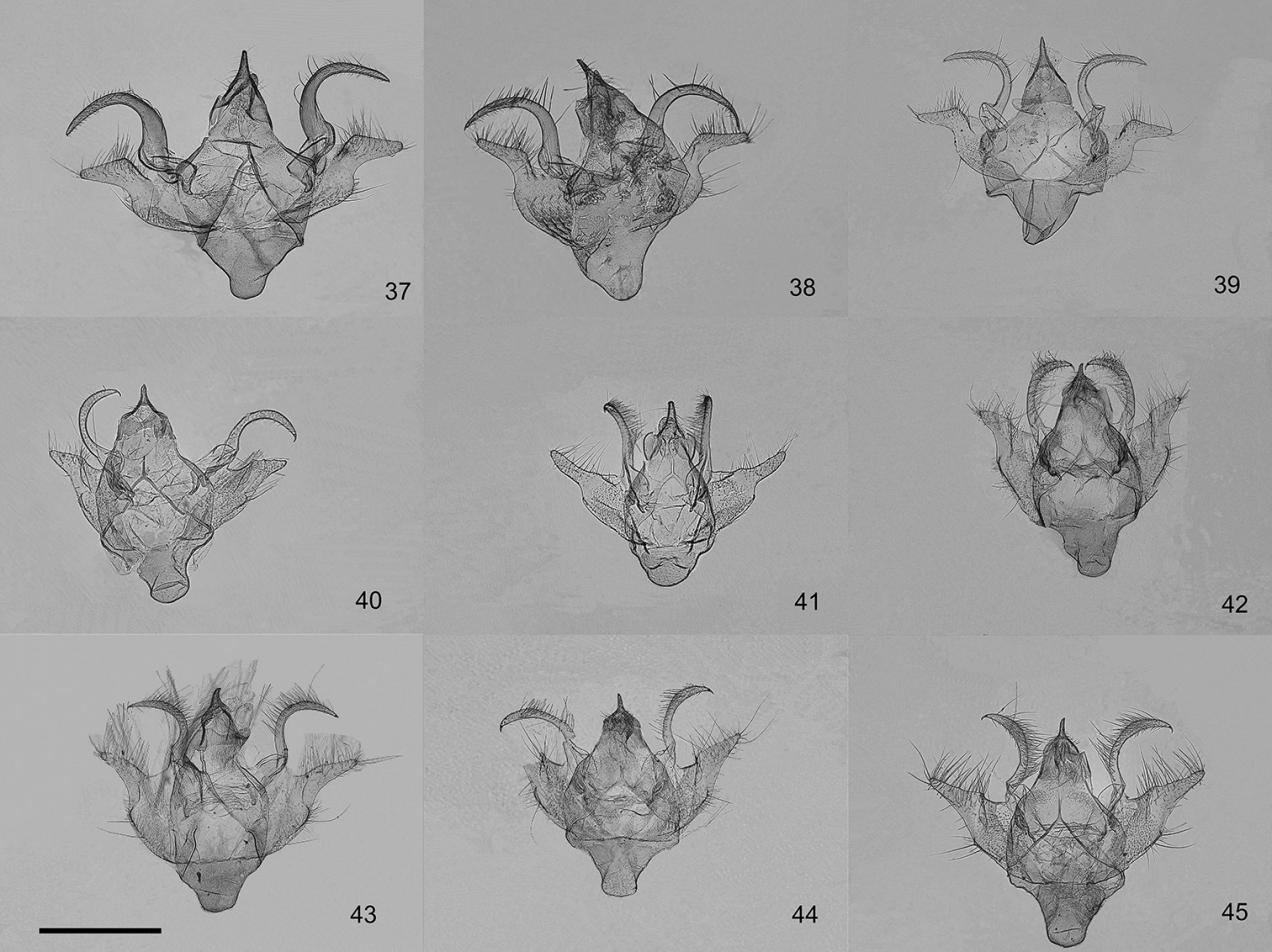
Male genitalia of the genus *Orbamia*. **37***Orbamia octomaculata***38***O. marginata***39***O. becki***40***O. renimacula***41***O. clarissima***42***O. clarior***43***O. obliqua***44***O. obliqua parva***45***O. ocellata*. Scale bar: 1 mm.

### Rabomia

Taxon classificationAnimaliaLepidopteraGeometridae

Hausmann & Tujuba gen. nov.

66BE934F-7E90-5427-B76C-FBF444B44109

http://zoobank.org/C6E24B77-0F95-457D-9034-26348A26F071

#### Type species.

Ectropis ? subaurata Warren, 1899.

#### Etymology.

The name is an anagram of the sister genus *Orbamia*, similarly to the anagram used by Herbulot (1966) when transforming the name *Boarmia* to *Orbamia*.

#### Differential features

(COI sequences, photographs of adults and their genitalia see https://dx.doi.org/10.5883/DS-ORBAMIA). Adult: Male antennae ciliate-fasciculate, female antennae filiform. Palpi of both sexes broad, bushy scaled, length in male 1.0, in female 1.5 times diameter of eye. Hind tibia of both sexes with two pairs of unequal spurs. Upperside of wings with inconspicuous, elongate discal spots, postmedial line of forewings sharp, strongly curved. Underside yellow, black terminal fascia conspicuous. Male genitalia: Uncus with three short sclerotised spines, lateral lobes below uncus swollen, saccus triangular, dorsal process of valva naked, with curved spinule at tip, aedeagus comparatively broad, with bundle of small cornuti at base, vesica condensed to a broad cornutus-like sclerite at tip. Female genitalia: Lamellae ante- and post-vaginalis sclerotised (elongate rhomboid), posteriorly rounded, ductus bursae sclerotised, long, helicoid, longitudinally furrowed, corpus bursae membranous, signum large, star-shaped.

#### Genetic data and phylogeny.

The maximum likelihood analysis of COI barcode data supports the monophyly of the genus *Rabomia* gen. nov. and sister group relationship with (*Pycnostega*+*Dorsifulcrum*) (cf. Table 1, Fig. 85). However, phylogenies at genus level need to be considered with caution, when they are inferred from COI data. More research is needed to investigate the potential (re-) assignment of *Dorsifulcrum* to the Cassymini after having been excluded from that tribe in Brehm et al. (2019).

#### Remarks.

In some morphological features (e.g., female signum and ductus bursae) the genus *Rabomia* gen. nov. is transitional to the genus *Dorsifulcrum* Herbulot, 1979, but is genetically clustering separately, underside of wings with black fascia only in the terminal area.

### Rabomia subaurata

Taxon classificationAnimaliaLepidopteraGeometridae

(Warren, 1899) comb. nov.

529648BB-D150-55E4-90CC-C314D66747D5

BIN: BOLD: AAM3217

Figures 15 , 33 , 51 , 69 , 82

Ectropis ? subaurata : Warren (1899): 306 (Holotype $ in NHMUK; locus typicus: [Zambia]: Mpeta, Loangwa River, off the Zambesi).

#### Examined material (ZSM).

10♂♀ from Zambia, Malawi, (southernmost) Democratic Republic of the Congo (Elisabethville [Lubumbashi]) (ZSM G 20923/♂; ZSM G20924 ♀).

#### Differential features

(COI sequences, photographs of adults and their genitalia see https://dx.doi.org/10.5883/DS-ORBAMIA): Adult: Forewing length: 13–14 mm. Upperside of wings: Ground colour comparatively dark grey. Underside: Ground colour yellow, discal spots small, terminal fascia narrow at centre of forewing termen interrupted by a large yellow area. Male genitalia: Valva long and narrow, sacculus narrowly projecting at tip, at the base of aedeagus a bundle of eight comparatively long (0.3 mm) microcornuti. Female genitalia: Star-shaped signum large, diameter 0.7–0.9 mm.

**Figures 46–54.**
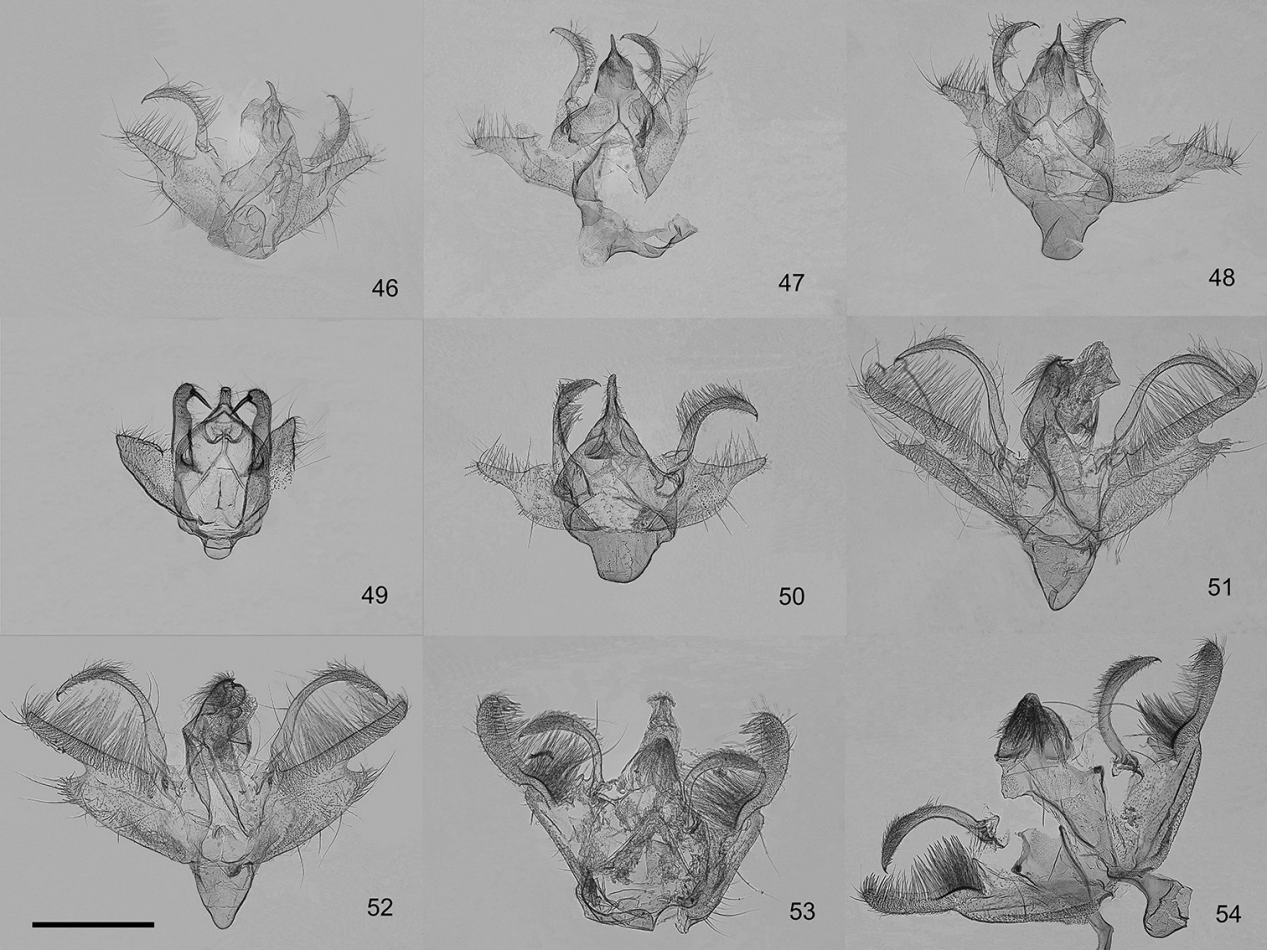
Male genitalia of the genera *Orbamia*, *Rabomia*, and *Morabia*. **46***Orbamia abiyi***47***O. emanai***48***O. emanai lenzi***49***O. pauperata***50***O. balensis***51***Rabomia subaurata***52***R. obscurior***53***Morabia politzari***54***M. brunnea*. Scale bar: 1 mm.

### Rabomia obscurior

Taxon classificationAnimaliaLepidopteraGeometridae

Hausmann & Tujuba sp. nov.

6EBC7E5E-4175-5B8F-A5EC-1E2E79EB98D6

http://zoobank.org/73A152CB-ADB8-4B67-AC34-A3F1BB1E16A2

BIN: BOLD: ABV9564

Figures 16 , 34 , 52 , 70 , 83

#### Examined material.

***Holotype***: ♀, Burkina Faso (Upper Volta), Bobo Dioulesso, 6.10.81, leg. Dr Politzar, coll. ZSM (G 20915).

***Paratypes***: 1♀, Burkina Faso (Upper Volta), Bobo Dioulesso, 13.11.85, leg. Dr Politzar; 1♀: Burkina Faso (Upper Volta), Bobo Folonso, 28.7.79, leg. Dr Politzar; 1♂, id., 10.11.74; 1♂, Nigeria, Kaduna, 3.vii.1970, leg. Politzar, coll. ZSM (G 20926); 1♀, id., 28.vi.1970; 1♀, id., 26.vi.1970; 1♀, id., 8.vii.1970; 1♂, North Nigeria, Kogin Kano Game Reserve, 15.vi.1974, leg. Dr Politzar; 1♀, Cameroon, Yala Yarna, 40 km N of Ngaoundéré, 735 m, 22.vii.1974, leg. Gilles Clément (ZSM G 20925).

#### Etymology.

The name refers to the darker colouration of wings (Lat. obscurior = darker).

#### Differential features

(COI sequences, photographs of adults and their genitalia see https://dx.doi.org/10.5883/DS-ORBAMIA): Adult: Forewing length: 12–14 mm. Upperside of wings: Ground colour with darker suffusion than in *R. subaurata*, medial fascia dark and conspicuous on all wings, sometimes anastomosing with postmedial line on forewing. Underside: Ground colour yellow, discal spots larger than in *R. subaurata*, terminal fascia much broader, at centre of forewing termen interrupted by a small yellow spot or even uninterrupted. Male genitalia: Valva long and narrow, sacculus edged at tip, only shortly projecting, at the base of aedeagus a bundle of twelve comparatively short (0.15–0.2 mm) microcornuti. Female genitalia: Star-shaped signum large, diameter 0.8–1.0 mm.

### Morabia

Taxon classificationAnimaliaLepidopteraGeometridae

Hausmann & Tujuba gen. nov.

D2ADFF75-42C8-5B92-AB67-A1ADE243CEF1

http://zoobank.org/D7BB6DF9-B1CD-4D00-8A15-A6906DFB2B56

#### Type species.

*Morabia politzari* Hausmann & Tujuba, sp. nov.

#### Etymology.

The name is an anagram of the sister genus *Orbamia*, similarly to the anagram used by Herbulot (1966) when transforming the name *Boarmia* to *Orbamia*.

#### Differential features

(COI sequences, photographs of adults and their genitalia see https://dx.doi.org/10.5883/DS-ORBAMIA). Adult: Male antennae ciliate-fasciculate, female antennae filiform. Palpi of both sexes broad, bushy scaled, length 1.0–1.5 times diameter of eye. Hind tibia of both sexes with two pairs of unequal spurs. Upperside of wings with discal spots vestigial, medial line zigzagging, terminal line conspicuous, zigzagging on all wings. Underside beige, with a few yellowish scales and a sharp black spot in forewing apex. Male genitalia: Uncus very short, rounded saccus shallowly projecting, dorsal process of valva strongly setose, valva long and narrow, curved, strongly setose, mainly at centre, aedeagus with long and stout cornutus. Female genitalia: Apophyses long and fine. Lamellae ante- and post-vaginalis membranous, ductus bursae straight, anteriorly membranous, posteriorly dilated and towards antrum strongly sclerotised, corpus bursae membranous, pyriform, signum absent.

#### Genetic data and phylogeny.

The maximum likelihood analysis of COI barcode data suggests the monophyly of the genus *Morabia* gen. nov. and an isolated position from (*Rabomia* gen. nov. (*Pycnostega*+*Dorsifulcrum*)) and from genus *Orbamia* (cf. Table 1, Fig. 85). However, phylogenies at genus level need to be considered with caution, when they are inferred from COI data.

**Figures 55–63.**
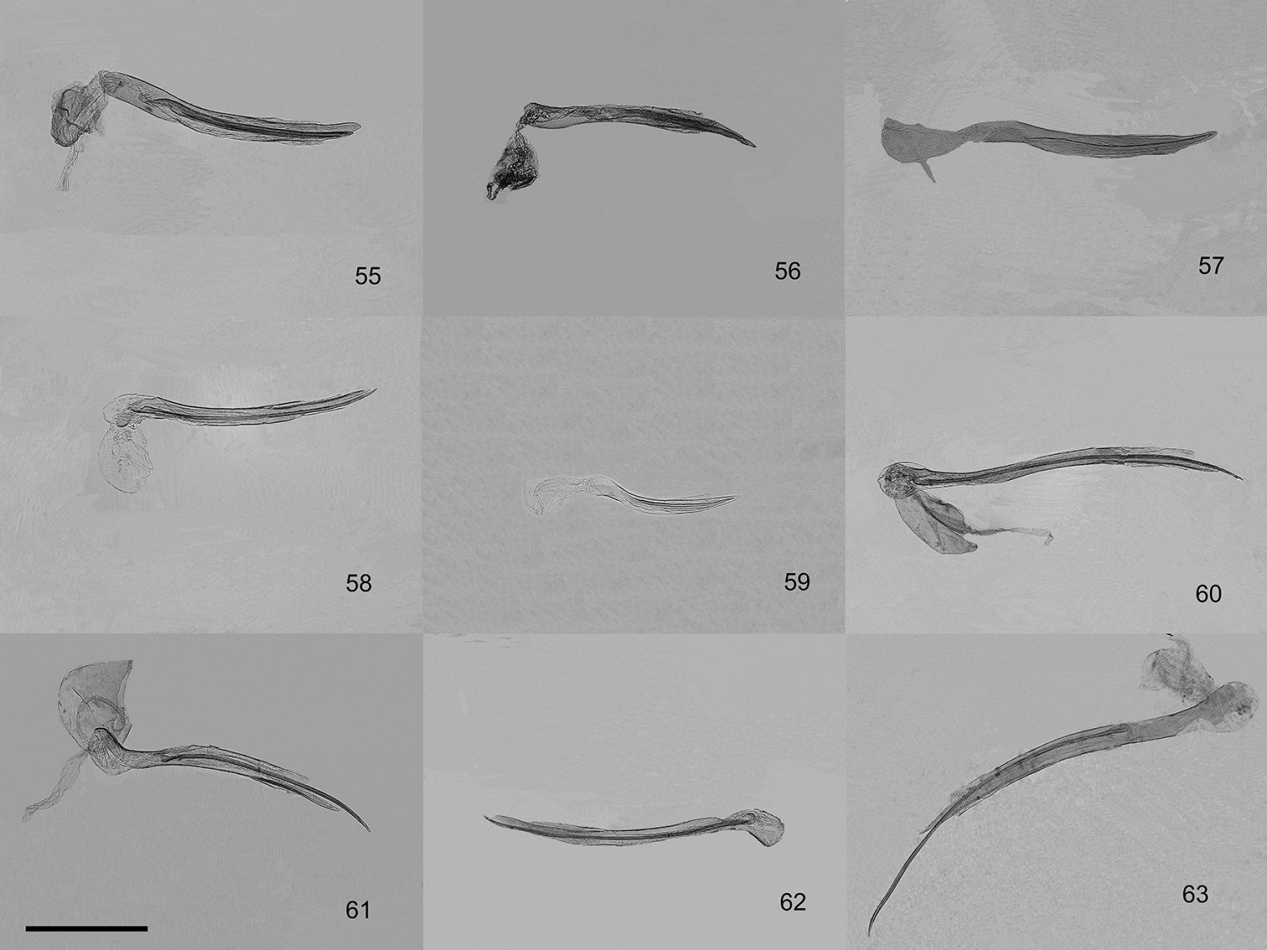
Aedeagus of male genitalia of the genus *Orbamia*. **55***Orbamia octomaculata***56***O. marginata***57***O. becki***58***O. renimacula***59***O. clarissima***60***O. clarior***61***O. obliqua***62***O. obliqua parva***63***O. ocellata*. Scale bar: 1 mm.

### Morabia politzari

Taxon classificationAnimaliaLepidopteraGeometridae

Hausmann & Tujuba sp. nov.

C0D633DE-4BD3-5A55-A4A5-6F03015B5471

http://zoobank.org/38C88B00-2394-4C29-81A9-9E58E06B058E

BIN: BOLD: ABX0432

Figures 17 , 35 , 53 , 71 , 84

#### Examined material.

***Holotype***: ♀, Kenya, Sokoke Forest, 31.7.94, leg. Dr. Politzar, coll. ZSM.

***Paratypes***: 1♂, Kenya, Watama, 2.viii.1973, leg. Politzar, coll. ZSM (G 20934); 1♀, id., 30.vii.1973; 1♀, Kenya, Sokoke Forest, 31. Vii. 1994, leg. Politzar; 1♀, id, 2.vii.1994; 3♀, Tanzanie Bagamoyo dist. Vigwaza, 231 m, 06°42.89'S, 038°52.52'E, 30.III 2014, leg. Ph. Darge; 1♀, Tanzanie, Pwani region, Ruvu forest reserve, 220 m, 06°57.27'S, 038°49.36'E, 03.III.2014, leg. Ph. Darge (ZSM); 1♀, Coll. Mus. Tervuren, E.ville [DR Congo], 16.X.1955, Seydel (ZSM G 20935).

#### Etymology.

The name refers to Dr. Heinz Politzar (1938–2007) for his great merits in collecting and studying African Lepidoptera (see Hacker and Hausmann 2010).

#### Differential features

(COI sequences, photographs of adults and their genitalia see https://dx.doi.org/10.5883/DS-ORBAMIA): Adult: Forewing length: 12–14.5 mm. Upperside of wings: Ground colour whitish beige. Underside: Ground beige, with slight yellow or orange tinge, mainly on forewing, discal spots small, both wings with sharp, black apical spot. Male genitalia: Uncus very short, rounded, saccus shallowly projecting, dorsal process of valva strongly setose, valva long and narrow, curved, strongly setose, mainly at centre, aedeagus (length 2.5 mm) with long and stout cornutus (1.9 mm), sigmoid at tip. Female genitalia: Lamellae ante- and post-vaginalis membranous, ductus bursae straight, anteriorly membranous, posteriorly dilated and towards antrum strongly sclerotised, corpus bursae membranous, pyriform, signum absent.

**Figures 64–72.**
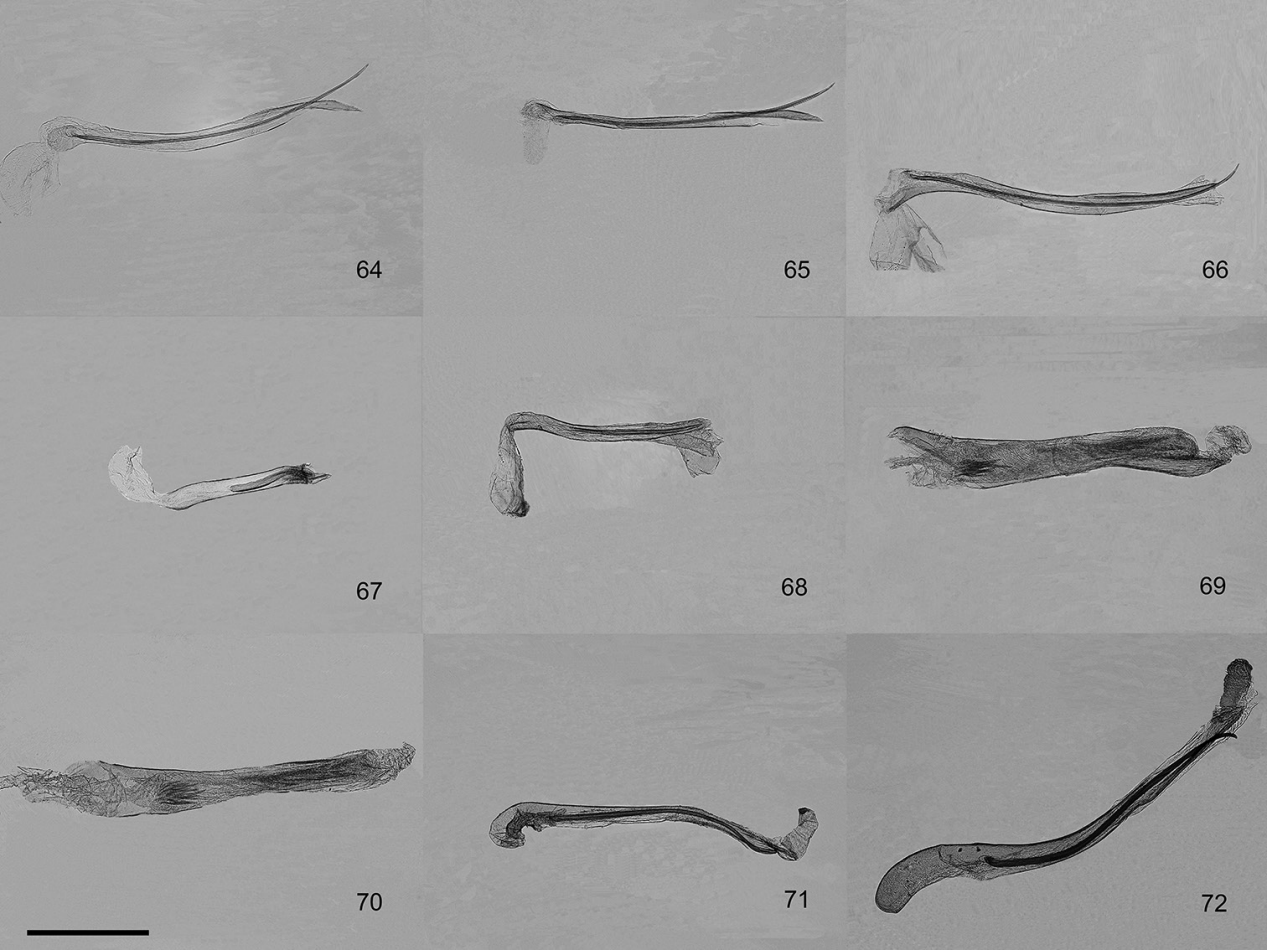
Aedeagus of male genitalia of the genera *Orbamia*, *Rabomia*, and *Morabia*. **64***Orbamia abiyi***65***O. emanai***66***O. emanai lenzi***67***O. pauperata***68***O. balensis***69***Rabomia subaurata***70***R. obscurior***71***Morabia politzari***72***M. brunnea*. Scale bar: 1 mm.

### Morabia brunnea

Taxon classificationAnimaliaLepidopteraGeometridae

Hausmann & Tujuba sp. nov.

B20955FD-0CCE-522D-8D81-C4E7469CE78E

http://zoobank.org/E25394ED-1400-454C-BDC5-882DAB89179C

BIN: BOLD: ABW6916

Figures 18 , 36 , 54 , 72

#### Examined material.

***Holotype***: 1♂, Zambia, North Zambia, Mutinondo, 1390 m, wet Miombo, 27.XII. 2010, 12°23'30"S, 31°19'23"E, light trap, J. Lenz legit, coll. ZSM (G 20945).

#### Etymology.

The name refers to the unusual, brownish ground colour of the wings (Lat. brunneus, -a, um = brown).

#### Differential features

(COI sequences, photographs of adults and their genitalia see https://dx.doi.org/10.5883/DS-ORBAMIA): Adult: Forewing length: 17.5 mm. Upperside of wings: Ground colour warm brown, pattern as in *M. politzari*. Underside: Ground pale brownish yellow, discal spots conspicuous, both wings with sharp, black apical spot and diffuse brown terminal fascia. Male genitalia: Uncus very short, rounded, strongly setose, saccus shallowly projecting, dorsal process of valva strongly setose, valva long and narrow, curved, strongly setose, mainly at centre, aedeagus longer (3.5 mm) than in the preceding species, S-shaped, with stout, S-shaped cornutus, longer than in the preceding species (2.3 mm). Female genitalia unknown.

**Figures 73–84.**
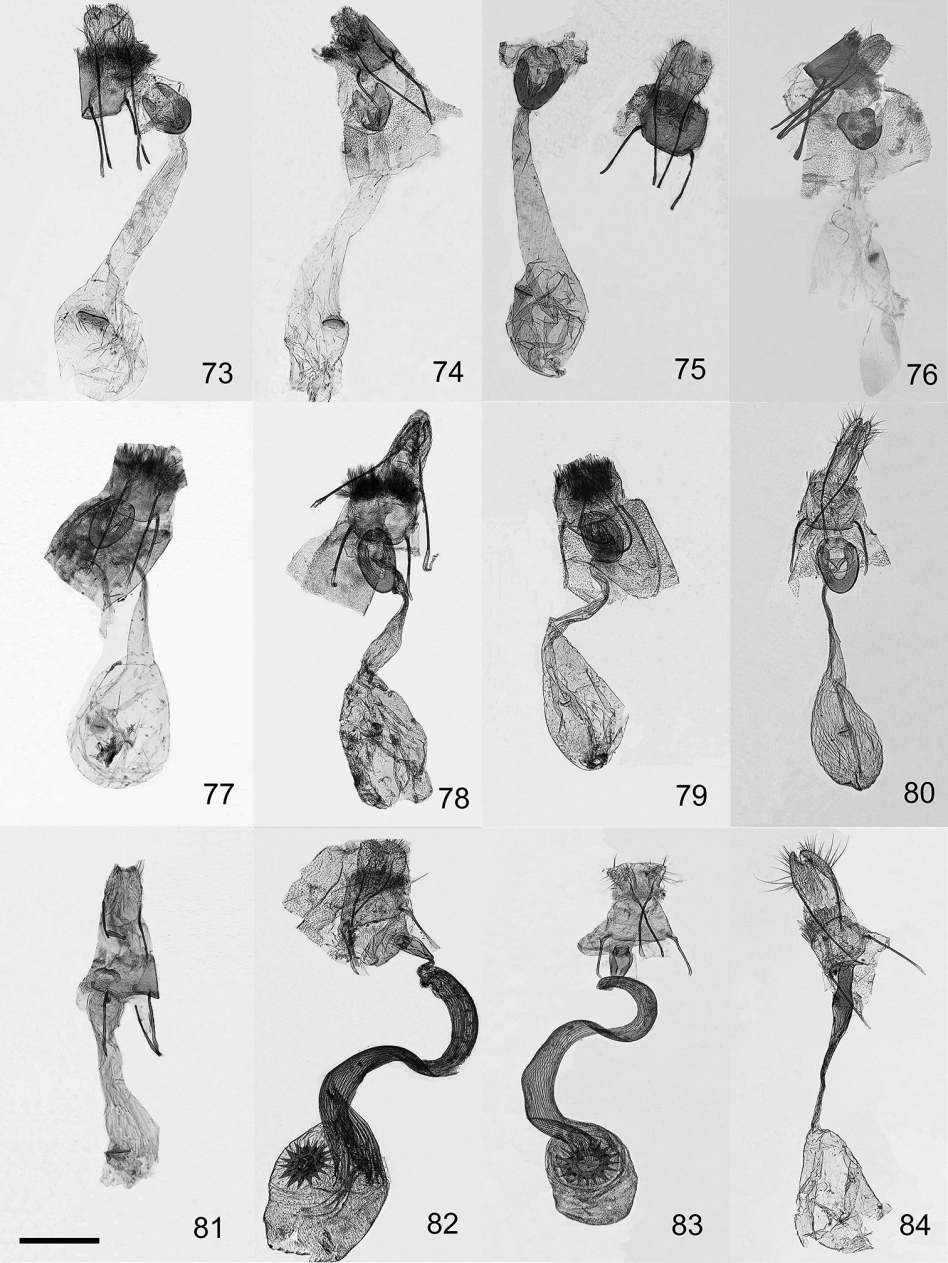
Female genitalia of the genera *Orbamia*, *Rabomia*, and *Morabia*. **73***Orbamia octomaculata***74***O. becki***75***O. renimacula***76***O. clarissima***77***O. ocellata***78***O. abiyi***79***O. emanai***80***O. emanai lenzi***81***O. pauperata***82***Rabomia subaurata***83***R. obscurior***84***Morabia politzari*. Scale bar: 1 mm.

**Figure 85.**
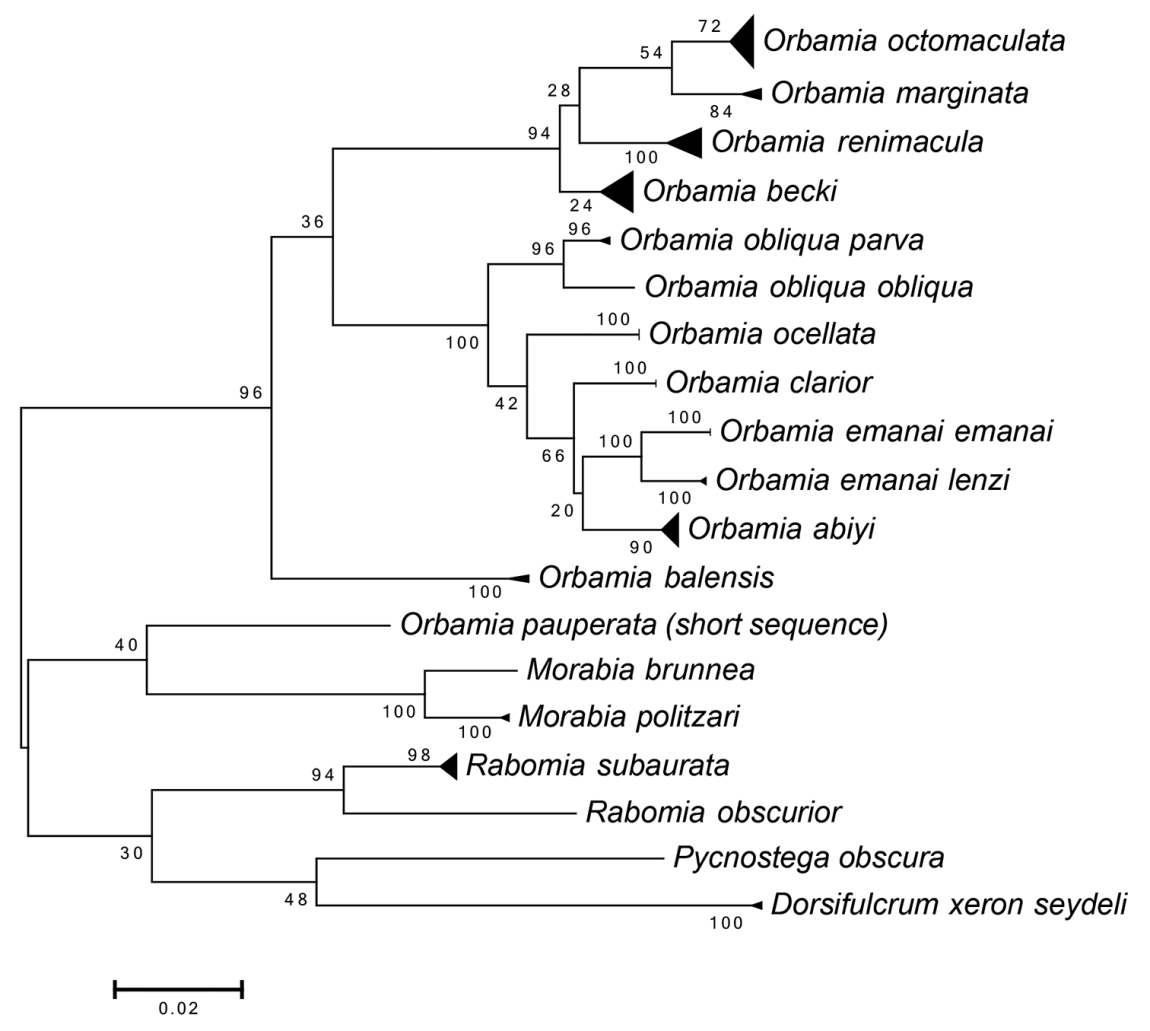
Maximum Likelihood Tree of COI data of the genera *Orbamia*, *Rabomia*, and *Morabia*, 50 Bootstrap Replications, Tamura-Nei model, uniform rates, built with Mega 6 software (Tamura et al. 2013; scale bar = 2 %), for original data see https://dx.doi.org/10.5883/DS-ORBAMIA.

## Supplementary Material

XML Treatment for Orbamia

XML Treatment for Orbamia octomaculata

XML Treatment for Orbamia marginata

XML Treatment for Orbamia becki

XML Treatment for Orbamia renimacula

XML Treatment for Orbamia clarissima

XML Treatment for Orbamia clarior

XML Treatment for Orbamia obliqua

XML Treatment for Orbamia obliqua parva

XML Treatment for Orbamia ocellata

XML Treatment for Orbamia abiyi

XML Treatment for Orbamia emanai

XML Treatment for Orbamia emanai lenzi

XML Treatment for Orbamia pauperata

XML Treatment for Orbamia balensis

XML Treatment for Rabomia

XML Treatment for Rabomia subaurata

XML Treatment for Rabomia obscurior

XML Treatment for Morabia

XML Treatment for Morabia politzari

XML Treatment for Morabia brunnea
